# Short vs. Long-Distance Avocado Supply Chains: Life Cycle Assessment Impact Associated to Transport and Effect of Fruit Origin and Supply Conditions Chain on Primary and Secondary Metabolites

**DOI:** 10.3390/foods11121807

**Published:** 2022-06-19

**Authors:** Romina Pedreschi, Excequel Ponce, Ignacia Hernández, Claudia Fuentealba, Antonio Urbina, Jose J. González-Fernández, Jose I. Hormaza, David Campos, Rosana Chirinos, Encarna Aguayo

**Affiliations:** 1 Facultad de Ciencias Agronómicas y de los Alimentos, Pontificia Universidad Católica de Valparaíso, Calle San Francisco s/n, La Palma, Quillota 2260000, Chile; excequel.ponce@pucv.cl (E.P.); ignacia.hernadez@pucv.cl (I.H.); claudia.fuentealba@pucv.cl (C.F.); 2 Department of Electronics, Computer Technology and Projects, Universidad Politécnica de Cartagena (UPCT), Plaza del Hospital 1, 30202 Cartagena, Murcia, Spain; antonio.urbina@upct.es; 3 Institute for Mediterranean and Subtropical Horticulture La Mayora (IHSM La Mayora-UMA-CSIC), 29750 Algarrobo-Costa, Málaga, Spain; jorgegonzalez-fernandez@eelm.csic.es (J.J.G.-F.); ihormaza@eelm.csic.es (J.I.H.); 4 Instituto de Biotecnología, Universidad Nacional Agraria La Molina (IBT-UNALM), Av. La Molina s/n, Lima 12056, Peru; dcampos@lamolina.edu.pe (D.C.); chiri@lamolina.edu.pe (R.C.); 5 Postharvest and Refrigeration Group and Quality and Health Group, Escuela Técnica Superior de Ingeniería Agronómica (ETSIA) and Institute of Plant Biotechnology, Universidad Politécnica de Cartagena (UPCT), Paseo Alfonso XIII, 48, 30203 Cartagena, Murcia, Spain

**Keywords:** life cycle analysis, Hass, fatty acid profile, tocopherols, phytosterols, phenolics

## Abstract

Avocado consumption and trade are increasing worldwide, with North America and Europe being the main importing regions. Spain is the major European avocado producer (90% of the production), yet it only supplies 10% of the market. Consequently, more than 90% of the avocados consumed in Europe are imported from overseas, mainly from Chile and Peru. In this work, the Life Cycle Assessment (LCA) impact associated with the transport of two avocado supply chains (short (Spanish) and long (Chilean)) and the effect of the fruit origin and distance of both chains on primary and secondary metabolites from harvest to edible ripeness were evaluated using a gas chromatography-mass spectrometry (GC-MS) and liquid chromatography coupled to diode array detection (LC-DAD) based metabolite analysis. The LCA transport impact of the fresh supply chain from production centers in Chile (Quillota) and Spain (Malaga), and then the distribution to several cities in Europe, suggested road export from Spain to European capitals to have the lowest impact (0.14 to 0.22 kg CO_2_ eq/kg of avocado). When export from Chile was considered, the option of oceanic freight to European ports closer to final destinations was clearly a better option (0.21 to 0.26 kg CO_2_ eq/kg) than via the Algeciras port in Spain followed by road transport to final destinations in European capitals (0.34 to 0.43 kg CO_2_ eq/kg), although the situation could be somewhat different if the avocados are transported from the destination ports in northern Europe to long-distance capitals in other European countries. Fruit origin had a significant impact on avocado primary and secondary metabolites. The conditions of the supply chain itself (10 d in cold storage in regular conditions vs. 30 d cold storage + controlled atmosphere conditions) largely influence the fate of some metabolites that certainly affect the pool of metabolites at edible ripeness. The long-assumed hypothesis that the longer the supply chain the more negative impact on nutritional and functional compounds might not hold in this case, as long as transport conditions are adequate in terms of temperature, atmosphere conditions, and time considering distance from origin to destination.

## 1. Introduction

The continuous increase in the consumption of fruits and vegetables has been associated with the prevention or mitigation of some chronic non-communicable diseases due to the presence of a wide range of secondary metabolites or phytochemicals [1]. Avocado is one of the fruits whose consumption has significantly increased in the last 10 years, with an average 15% per year increase mainly due to its appreciated taste, texture, and culinary characteristics [2,3] besides the presence in its composition of primary (mannoheptulose and perseitol, fatty acid profile, tocopherols) and secondary (phytosterols, phenolic compounds, carotenoids) metabolites that confer this fruit a wide range of positive nutritional and functional properties [4,5].

Most of the avocado international market worldwide relies on a single variety, ‘Hass’, which originated in California almost 100 years ago [6]. South American countries such as Chile and Peru are currently the main avocado suppliers for the European market. Spain is still by far the main European country with a significant avocado production (61,000 t in the 2018/2019 season) according to the Agrarian Association of Young Farmers [7] with the main advantage being that the fruit reaches the consumers in the European market in a few days compared to the several weeks of the avocados produced in South America. Spanish production is concentrated in the provinces of Malaga and Granada. In recent years, an expansion of the areas where avocado is grown in Europe has been taking place; not only in Spain (western Andalusia and the Valencia areas) but also in Portugal (mainly in the Algarve region) and Italy (Sicily). However, avocado production in continental Europe represents less than 10% of the European consumption and, consequently, it is insufficient to satisfy the increasing European market demand, which substantially relies on international commerce supply by different origins [5]. 

The sustainability of avocado production is being questioned due to its water and carbon footprints as well as its social impacts [8]. For the first time, the environmental impacts (e.g., primary energy demand, water footprint, global warming potential, land use including natural and land transformation and urban land occupation, fossil depletion, ecotoxicity, ozone depletion, among others) of different fruits consumed in UK have been reported. In this study, avocado ranked among the worst fruits in terms of environmental impacts, displaying the highest agricultural land use, water footprint, marine eutrophication, and human toxicity [6]. Most of the global warming (GWP) potential corresponded to the farm processes (~42% of total GWP) but transport also had a significant contribution in this indicator (~40% of total GWP) [8]. However, avocado is grown in about 50 countries, and the problems and impacts of its cultivation that affect its sustainability depend on the growing regions; nevertheless, since this fruit is considered a commodity, particular problems in a specific region of the world are often translated to avocado worldwide. The sustainability of the avocado production and commercialization should thus be analyzed in a case-by-case basis.

On the other hand, environmental sustainability must be in line with a healthy and nutritional overall status [9,10]. According to Clark and Tilman [11], fruits, including avocado, have an environmental impact up to 100 times lower and are also important sources of nutrients (e.g., vitamins) compared to ruminant meat and other food products such as milk, eggs, pork, poultry, and seafood. Avocado is considered a highly nutritious fruit and contains most of the compounds [6] entailed in the Nutrient Rich Foods Index NRD 9.3 (protein, dietary fibre, calcium, iron, potassium, magnesium, and vitamin A, C, and E, and exclusion of saturated fat, added sugar, and sodium) [12] and the EAT-Lancet Commission on healthy diets [13].

Important, efforts are being taken to increase water productivity (crop yield per volume of water supplied by irrigation) and water use efficiency (controlled deficit irrigation) in the different avocado producing countries threatened by climate change effects [14,15], including more efficient cultivation systems [16]. These efforts should be reflected in lower water and carbon footprints at the farming stage for avocado production. Additionally, according to Poore and Nemecek [17], 17 kg out of 100 kg of food produced is transported internationally, increasing to 50 kg for nuts and 56 kg for oil. Since most of the avocados consumed in Europe (~90%) are imported from outside the continent, it is important to assess the carbon footprint of avocado associated with transport in different commercialization scenarios.

Only one study has reported on the differences in primary metabolites (e.g., fatty acid profile and C7 sugars) in avocado cv. Hass from different origins (Spain, Chile, and Peru), considering the different growing locations and commercialization chains [18]. In this study, Spanish and Chilean Hass avocados showed a higher percentage content of oleic acid (~60%) compared to Peruvian avocados (~45%). Recent studies have reported the evolution of fatty acids and secondary metabolites (e.g., carotenoids, tocopherols, phytosterols, and phenolic compounds) of Hass avocados, simulating air storage conditions for prolonged times [19], prolonged cold storage in controlled atmosphere simulating international transport [20], or subjected to ripening up to 14 d at 20 °C [21]. These studies have reported that, in general, major phytochemicals (phenolics) tend to increase as ripening proceeds. 

The most complete reported work on LCA analysis (from production to consumption) for different fruits consumed in UK was reported by Frankowska et al. [8]. Another recent research work on Chilean apple LCA analysis destined to European markets revealed that ocean freight accounts for 39.2% of the carbon footprint [22]. A review of the literature shows that there are no previous studies dealing with the impact of both fruit origin and distance, including conditions of the commercialization supply chain, of avocado cv. Hass on environmental and human health indicators or on primary and secondary metabolites of nutritional and functional interest. Thus, the aims of this research were to evaluate: (i) the Life Cycle Assessment (LCA) impact associated to the transport of two different distance avocado supply chains (short (Spanish) and long (Chilean)) and (ii) the effect of both fruit origin and conditions of these two commercially representative chains on primary and secondary metabolism from harvest to edible ripeness.

## 2. Materials and Methods

### 2.1. Fruit Material and Simulation of Shipping Conditions

Two hundred avocado fruits cv. Hass of export quality (~250 g) were sourced from the IHSM La Mayora, Malaga, Spain, and a similar number of fruits and weight were sourced from the Region of Valparaiso, Chile during the season 2019/2020. Avocado cv. Hass from both countries represent short (Spain) and long-distance (Chile) supply chains. Growing conditions in terms climatic conditions correspond both to Mediterranean type conditions. For Chilean Hass avocados, the fruit was collected from an orchard located from the interior zone, which has an average temperature growth of 14.2 °C (Hernández et al., 2021). Spanish Hass avocados from La Mayora have an average temperature growth of 19.4 °C. Sampling considered fruits displaying similar dry matter contents (>23–26%), which represents the similar fruit developmental stages for both origins. Dry matter content is the main criterion to start avocado harvesting. The simulation of the short distance supply chain involved 10 d of storage in regular air conditions at 5 °C followed by shelf-life conditions at 20 °C and 65–70% RH until the ready-to-eat stage (4–14 N) was attained (~10–15 d). Simulation of the long-distance supply chain involved 30 d storage in controlled atmosphere conditions (4 kPa O_2_ and 6 kPa CO_2_ at 5 °C for simulation of export market from Chile to Europe) followed by shelf-life conditions at 20 °C and 65–70% RH until the ready-to-eat stage (4–14 N) was attained. CA conditions were generated in a controlled atmosphere system (Happy Volt, Santiago, Chile) composed of individual 150 L chambers with automatic control of oxygen and dioxide concentrations. The CA system is placed inside a cold room that allows for temperature control and monitoring. 

### 2.2. Assessment of the Environmental Impact

One of the goals of this study is to evaluate the LCA impact of Hass avocado with focus on the transport stage of the fresh supply chain from production centers in Chile (Quillota) and Spain (Malaga) and then distributed to several cities in Europe. A comparative LCA methodology has been applied to analyze the impacts of different options for the avocado transport. The method followed ISO 14040 [23] recommendations and used ReCiPe v1.12 [24], both at midpoint (natural environmental aspects) and endpoint (damage effect on the environmental aspects), for life cycle impact assessment for a functional unit (FU) of the transport required for 1 kg of avocado from the production location to the final location of central market distribution in several European cities. As midpoint impact categories, climate change and fossil resource scarcity were selected. The midpoint characterization factor selected for climate change is the widely used global warming potential (GWP), which quantifies the integrated infrared radiative forcing increase of a greenhouse gas emission (GHG), expressed in kg CO_2_eq [25,26]. The use of a quantified kg CO_2_eq impact category enables a fair comparison with other transport means and routes. The midpoint indicator for fossil resource use, determined as the Fossil Fuel Potential (FFP in kg oil-eq), is defined as the ratio between the higher heating value of a fossil resource and the energy content of crude oil [27]. The damage assessment pathways considered to travel from the midpoint to the endpoint level are grouped in three categories: resources depletion or scarcity, human health, and ecosystem quality. The unit for human heath damage, DALYs (disability adjusted life years) measures the aggregated burden of human disease. The impact on terrestrial, freshwater, and marine ecosystems, measured in species per year, describes aggregated absolute species loss at the local, regional, and global scale. The unit for resource scarcity is in USD dollars ($) and represents the extra costs involved for future mineral and fossil resource extraction. 

Cumulative primary energy demand (CED v1.8) [28], is another method that has also been used to compare the different transport options. This method calculates the direct and indirect energy used throughout the life cycle of a product and differentiates among renewable and non-renewable energy sources, allowing to calculate the environmental effects related not only to the emissions but also to the consumption of energy [29]. In our study, the CED was calculated by including both non-renewable (from fossil fuels, nuclear, and nonrenewable biomass) and renewable (from wind, solar, geothermal, and water) energy sources. Ecoinvent v3.8 [30] was accessed and software SimaPro v9.1.1 was used for the calculations [23,31]. 

The two mentioned production centers (Quillota and Malaga) were selected as representative of similar locations in both Chile and Spain, respectively, in which a local road transport in origin was included in the LCA study. In the case of Chile, travel was considered as comprising local transport (a lorry between 16 and 32 metric tonnes), then an oceanic freight from San Antonio port to either Algeciras port in Spain or other European ports and followed by the required final road transport to market distribution locations (in this case a lorry larger than 32 tonnes, not including final retail delivery). Temperature during the whole transport corresponded in both cases to 5 °C and represent real life conditions. The oceanic freight was calculated in km using the information about commercial routes in Ports website, available online: http://www.ports.com (accessed on 15 January 2020) [32]. Distances covered by all terrestrial transports were determined using Google Maps [33]. To obtain a more direct comparison of transport alternatives, the distribution of Chilean avocado exported via Algeciras was carried out by road transport to Malaga, where there is a redistribution center, and then to the final destinations in Europe. Alternative arrival at other European ports (Rotterdam, Portsmouth, and Marseille) has been added to the comparative study, which was then followed by shorter road routes to the respective country capital cities. The distances considered for the calculation are indicated in Table 1 and comprise three stages in the case of Chile: from the origin to the main port of export, from the port of export to the different import ports in Europe, and from the import ports to different capital cities in Europe by road. In the case of Spain, only road travel from the origin to the different capital cities in Europe is considered. The packaging considered is that which is most used for avocado—cardboard boxes (40 × 30 × 9.5 cm) with a standard weight of 4 kg. For overseas transport from Chile, 1 × 1.20 m wooden pallets are used, in which 264 boxes per pallet are loaded. A controlled atmosphere and refrigerated 40-foot container, with a full load of avocado (22 pallets) are used to transport the fresh fruit from Chile to Europe. On the return trip, the usual practice is to load the containers and trucks with other foods and, thus, only distance traveled in one direction of the journey was included. Details of this transport are presented in Weidema et al. [34]. For road transport from Spain, 0.8 × 1.2 m. europallets are used with 176 boxes, 33 pallets, and a total of 23.232 kg per refrigerated truck.

### 2.3. Fruit Quality Evaluations

Dry matter content was determined at 100 °C × 24 h (constant weight attained) for both Spanish and Chilean Hass avocados based on 20 independent fruits from each origin. Fifty fruits were independently analyzed at harvest for firmness and internal and external quality evaluations as detailed in Rivera et al. [35]. Internal evaluations included pulp and vascular browning, anthracnose and stem end rot and external evaluations included incidence of black spot, lenticel damage and russet. After simulation of both chains (short and long-distance), 50 fruits were analyzed for quality assessment and finally 50 fruits were analyzed at the ready-to-eat stage (4–8 N) for internal and external quality attributes. Similar hedonic scale from 1 to 5, as in Ref. [35], was used to assess the different internal and external quality evaluations: 1 = no occurrence; 2 = slight damage; 3 = moderate damage; 4 = moderately severe damage; 5 = severe damage.

### 2.4. Assessment of Primary and Secondary Metabolites

Main primary (fatty acids, sugars, organic acids, and amino acids) and secondary metabolites (total phenolic compounds and profile and in vitro hydrophilic and lipophilic antioxidant activity) were analyzed at harvest, after simulation of shipping conditions (short vs. long-distance supply chains) and at edible ripeness after simulation of chain conditions. A total of 10 independent fruits were analyzed per sampling point. 

#### 2.4.1. Determination of Fatty Acids

Fatty acids were determined according to the protocol described by Campos et al. [19] using the methylation protocol of Meurens et al. [36] and the same chromatographic conditions. FAMEs were identified and quantified based on retention time comparison with previously injected external standards. The results were expressed as g fatty acid kg^−1^ oil.

#### 2.4.2. Determination of Polar Metabolites

Relative quantification of sugars, organic acids, amino acids, and other polar metabolites were determined according to the protocol of Hatoum et al. [37], slightly modified by Fuentealba et al. [38], based on gas chromatography mass spectrometry analysis. The main sugars, organic acids, and amino acids were quantified by using calibration curves built with external standards. Results were expressed as g metabolite kg^−1^ DW. Other polar metabolites were reported based on relative quantifications.

#### 2.4.3. Determination of Phenolic Compounds

Phenolics extracts were obtained following the protocol described by Uarrota et al. [39]. Polyphenolic compounds were analyzed using an Ultra High Performance Liquid Chromatography (UPLC) system composed of an Acquity H Class separation module (Waters, MI, USA) equipped with an auto-injector, an Acquity photodiode array detector (PDA eλ detector), and the Empower software. The column used for UPLC separation was an Acquity BEH C 18 (1.7 μm, 100 × 2.1 mm) (Waters, MI, USA) with an Acquity Vand Guard BEH C 18 pre-column (1.7 μm, 5 × 2.1 mm), operated at 30 °C. The mobile phase consisted of 0.1% formic acid in water (solvent A) and 0.1% formic acid in acetonitrile (solvent B). The gradient was as follows: 2% B for 2 min, 2 to 7% B in 2 min, 7 to 12% B in 11 min, 12 to 26% B in 5 min, 26 to 55% B in 5 min, 55 to 9 5% B in 1 min, and 95% B for 3 min. The flow rate and sample injection used were 0.2 mL min^−1^ and 2.0 µL, respectively. Spectral data were recorded from 200 nm to 700 nm during the whole run. Phenolic compounds were identified and quantified by comparing their retention time and UV-visible spectral data to known previously injected standards (280, 320, and 360 nm). The results were expressed in g kg^−1^ DW.

#### 2.4.4. Determination of Abscisic Acid Content by UPLC-PAD

Abscisic acid (ABA) was quantified from the same extract used for the analysis of hydrophilic antioxidant capacity (H-AC). ABA was quantified using the UPLC Aquity H-Class (Waters) system coupled to a PDA detector (eλ detector) and Empower II software. An Acquity BEH C18 column (1.7 µm, 100 mm × 2.1 mm) (Waters) with a BEH C18 column guard (1.7 µm, 5 mm × 2.1 mm) was used. The mobile phase was composed of (A) 0.1% formic acid in MilliQ water and (B) acetonitrile with 0.1% formic acid. The gradient used was as follows: 2% of B for 2 min, 2–7% of B in 2 min, 7–12% of B in 11 min, 12–26% of B in 5 min, 26–55% of B in 5 min, and 95% B for 3 min, and the column was equilibrated with 2% B for 5 min. The injected volume was 10 μL, with a flow of 0.2 mL min^−1^, and a column temperature of 30 °C. ABA was identified and quantified by comparing its retention time and UV-visible spectral data to previously known injected standard (260 nm). The results were expressed in g kg^−1^ DM.

#### 2.4.5. Determination of In Vitro Hydrophilic and Lipophilic Antioxidant Capacity

The ABTS assay was used for the determination of the in vitro hydrophilic and lipophilic antioxidant capacity (HAC and LAC) as previously described by Arnao et al. [40] with slight modifications. The antioxidant capacity was calculated as mmol of Trolox equivalents (TE) kg^−1^ DM from a standard curve developed with Trolox.

### 2.5. Statistical Analysis

One way analysis of variance (ANOVA) followed by Tukey’s test for multiple comparisons (*p* < 0.05) were performed using Statgraphics 18 (StatPoint Inc., Rockville, MD, USA). The results were expressed as means ± standard deviation. 

## 3. Results and Discussion

### 3.1. Differences in Environmental Impacts between Short and Long-Distance Supply Chains

**Figure 1:**
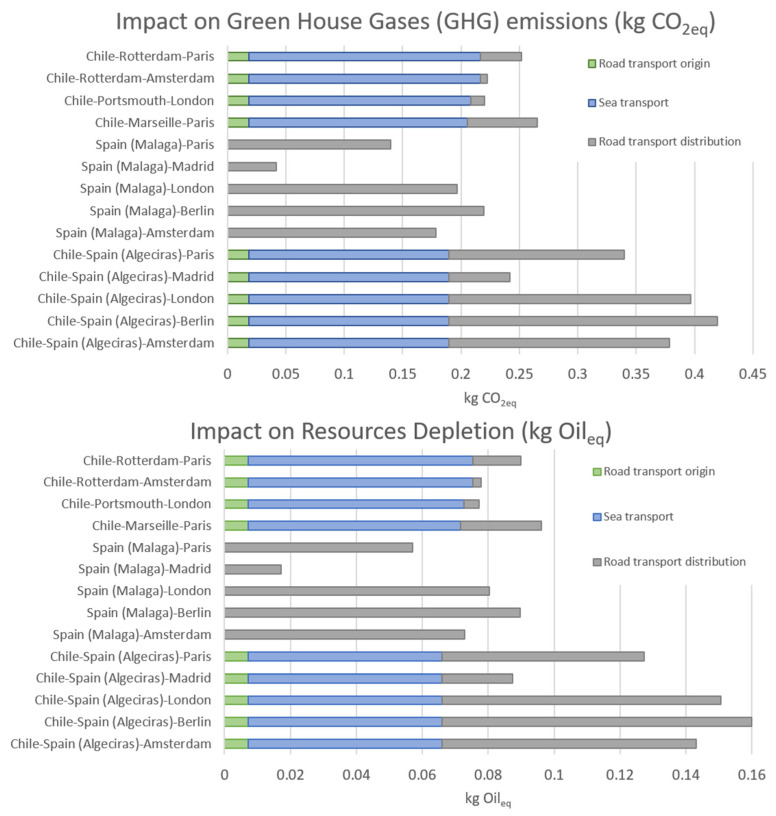
Impacts on two midpoint categories of different options for the transport of 1 kg of avocados, indicating the contribution of the three parts of the total trip: road transport at origin (green), oceanic freight (blue), and final road transport to distribution markets in Europe (grey). The two categories presented are Green House Gases (GHG) emissions (kg CO_2_eq) and Resources Depletion (kg Oileq).

According to the ReCiPe v1.12 methodology [24], the corresponding impacts at two midpoint categories, GHG emissions (measured in CO_2_eq) and resources depletion (measured in kg Oileq), are shown in Figure 1. 

In both cases the contribution of the three supply chains (Chilean or European road and sea transport) of the freight are indicated by different colors in the stacked bars. As expected, for both the GHG emissions and resource depletion impacts the overseas transport presented a higher impact than the European internal road transport since, in this case, trips by oceanic freight are much longer than road trips. In our case, the maritime transport, from port (Chile) to port (Europe), presented an average of 0.17 to 0.20 CO_2_ eq/kg of avocado considering a direct non-stop trip that was fully loaded and returned with other foods. The route with the highest GHG emission was Chile to Germany (Berlin) through Algeciras port (Spain), which presented 0.42 CO_2_ eq/kg versus a direct road trip from Spain (Malaga) to Berlin with 0.21 CO_2_ eq/kg. The use of ports closer to the final destination significantly reduces impacts by decreasing road transport in Europe. However, it should be considered that the situation will be different if the avocados are transported from the destination ports in northern Europe to long-distance capitals in other European countries, since this will increase emissions due to long road transport. This is very important since more than 50% of the avocados consumed in Europe are re-distributed from the Netherlands [41] to the rest of the continent and CO_2_ emissions will increase since usually road transport is used. Similar studies have been performed in other fruits. For example, for banana from Ecuador, Roibás et al. [42] reported 0.23 kg CO_2_ eq/kg banana for overseas transport from Puerto Bolivar and Guayaquil harbors in Ecuador to Rotterdam (Netherlands). Chilean apple presents GHG emissions of 0.54 kg CO_2_ eq/kg, being the ocean freight from Chile (Valparaiso) to UK (London), a hot spot (0.21 kg CO_2_ eq/kg) that determined the performance of the product carbon footprint of exported apple with a contribution of 39.2% [22]. This result is similar to that reported here for the route Chile (Quillota) to UK (London) for avocado.

**Figure 2:**
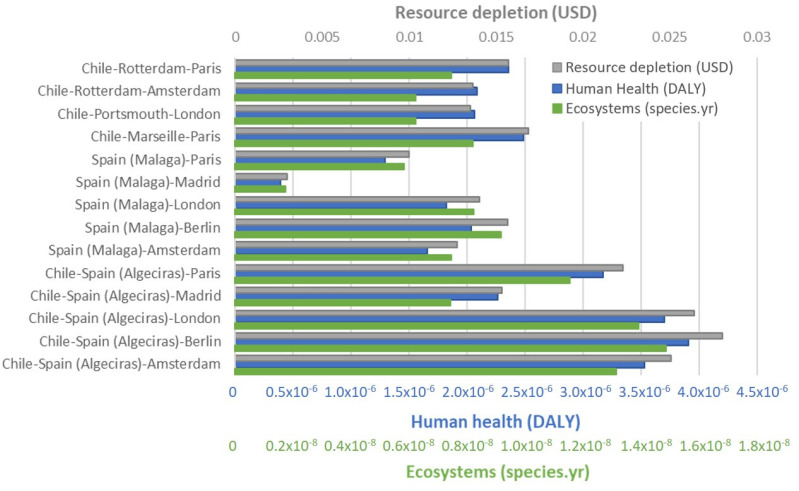
Impacts on three endpoint aggregated categories: Resource depletion (USD, grey), Human Health (DALY, blue), and Ecosystems (species year, green) for the different transport route options for exporting 1 kg of avocados from Chile and Spain to several European cities.

**Figure 3:**
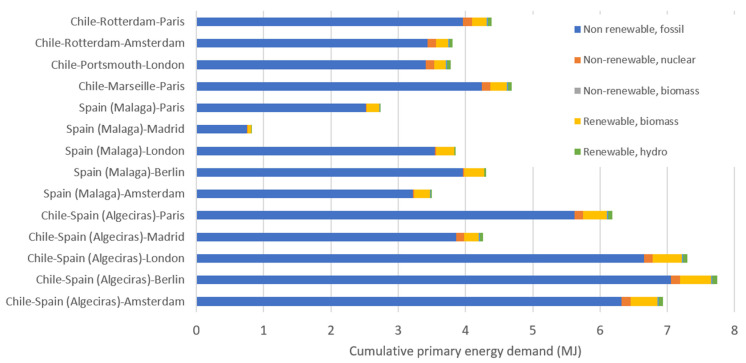
Cumulative primary energy demand (MJ) for the different routes of transport of 1 kg of avocados. Chile and Spain are considered as the travel origin; for Chile two options are considered: (i) export via Algeciras port and then using the same route as Spain exports (lower five bars) and (ii) export via Rotterdam, Portsmouth, or Marseille (as indicated in the four upper bars) and the road to the respective city.

One interesting observation reported in this paper is the three groups of endpoint categories quantified in Figure 2. ReCiPe endpoint calculation delivers a quantitative result for resources depletion (USD dollars), human health (DALY), and impact on ecosystems (species per year). National avocado road transport in Spain (from Malaga to the capital, Madrid, with a distance of 539 kms) provided the lowest impact compared to the combination of overseas transport and long road distance. According to the CED method, the different transport options of 1 kg of avocado are shown in Figure 3. As mentioned before, the national transport in Spain is the option with lower CED (0.82 MJ), followed by export from Spain by road to other European capitals with values between 3.5 MJ and 4.3 MJ. When export from Chile is considered, the option of oceanic freight to European ports closer to final destinations is clearly better with values between 3.8 MJ to 4.4 MJ than exporting Chilean avocados via Algeciras port, when CED values jump to values around seven MJ.

Following the trend envisaged with CED analysis, as expected for a LCA focused on transport, the impact of the different route options confirms that lower impacts are obtained for avocado exported from Spain, followed by Chilean exports via ports closer to final destination cities and finally Chilean exports via Algeciras and then large road trips in Europe. Both in national and maritime transport, the non-renewable fossil energy as production and consumption of fuel oil, was the major contributor to the CED.

### 3.2. Differences in Internal and External Quality Attributes in Hass Avocado Fruits from Different Origins and Supply Chain Conditions

To establish fair comparisons between fruit origin at harvest and onwards (supply chain), given that the minimum harvest maturity index of Hass avocado for Spain and Chile are different (21% and 23%, respectively), in this study Hass avocados within a dry matter (DM) content range of >23–26% were evaluated. The Spanish and Chilean avocados analyzed presented an average dry matter content of 25.9 ± 2.9% and 24.7 ± 2.5%, respectively. Previous studies have determined that as harvest time advances during the season, dry matter increases and differences in primary and secondary metabolites can be observed [19,43]. Thus, this standardization based on dry matter content is necessary to make appropriate comparisons between different producing regions.

Non-destructive firmness measurements were carried out at harvest, after the simulation of the supply chain (short and long) and at the ready-to-eat stage with values for Spanish and Chilean avocados corresponding to 121.3 ± 12.1 N, 106.2 ± 18.8 N and 10.9 ± 1.4 N (short supply chain) and 88.2 ± 14.8 N, 76.7 ± 14.5 and 8.6 ± 3.9 (long-supply chain), respectively. No external disorders (russet, brown spots due to cold damage) and internal disorders (vascular and pulp browning) were observed for fruits of both origins from harvest to edible ripeness.

### 3.3. Differences in Primary Metabolites in Hass Avocado Fruits from Different Origins and Distance and Condition Supply Chains

Avocado cv. Hass is a rich source of nutrients, especially its high oil content characterized by the presence of unsaturated and polyunsaturated fatty acids, oleic acid being the predominant one. Average oil contents at harvest of 11.8 and 10.1% were obtained for Spanish and Chilean Hass avocados, respectively (Table 2). The apparent increases observed after the short and long-distance postharvest transport conditions and at the ready-to-eat stages are due to water loss and the easiness to extract oil as maturity advances during transport simulation and shelf-life conditions. 

This is more pronounced for avocados from Spain since simulation of transport/storage conditions were carried out in refrigerated conditions at 5 °C while avocados from Chile were stored in controlled atmosphere conditions of 4 kPa O_2_ and 6 kPa CO_2_ at 5 °C for 30 d. Previous studies have reported that avocado oil is only biosynthesized while the fruits are on the tree and that the apparent increases during postharvest storage and ripening are due to water loss and the easiness to extract the oil [43]. More interesting were the differences in the fatty acid profiles between avocados cv. Hass from Chile and Spain, with very similar dry matter and oil content values. Differences in the amounts of different fatty acids between avocados from Spain and Chile were also found (Table 2). Oleic acid, the main unsaturated fatty acid in Hass avocado, was significantly higher in Spanish avocados, representing 51.1% of the total fatty acids versus only 43.5% in Chilean Hass avocados. Other polyunsaturated fatty acids such as linoleic (13.5% and 16.9%) and α-linolenic (0.2% and 1.5%) were lower in Spanish than Chilean avocados. The fatty acid profiles and percentages did not change during the whole supply chain as previously reported [43]. Differences were related to fruit origin and associated to the different growing conditions, especially differences in temperature fluctuations during fruit growth and development, which result in a longer period from flower to harvest in Chile than in Spain [16,19,43,44]. Donetti and Terry [18] and Pedreschi et al. [43] reported a much higher oleic acid content in Chilean Hass avocados than those shown in this study. Differences could likely be attributed to the orchard specific climatic conditions where avocados were sourced in each study.

C7 sugars (mannoheptulose and perseitol) are the main soluble and respiratory sugars in avocado, followed by the C6 sugars (sucrose, glucose, and fructose) representing in total up to 97% of total soluble sugars in avocado [4]. In this study, we only carried out a quantification of these sugars in Hass avocado from Spain and Chile at the same maturity stage and simulating their supply chain conditions (short and long). Differences in abundance of perseitol, mannoheptulose, and sucrose were found at harvest between the fruits from both origins. A lower content of perseitol (45.4 g kg^−1^ DW) but a higher content of mannoheptulose (84.7 g kg^−1^ DW) was found in Spanish avocados compared to Chilean avocados (74.9 g kg^−1^ DW and 33.12 g kg^−1^ DW of perseitol and mannoheptulose, respectively). The different supply chain scenarios short vs. long (10 d at 6 °C and 30 d at 5 °C and 4 kPa O_2_ and 6 kPa CO_2_) did not affect the content of perseitol. However, mannoheptulose decreased in the short distance supply chain (from 84.7 to 50.5 g kg^−1^ DW) indicating that there is a significant on-going metabolic activity in regular air conditions compared to the long-distance supply chain (lower temperature and controlled atmosphere). Significant on-going metabolic activity in cold regular air conditions compared to controlled atmosphere conditions is well noted since under regular air conditions the fruit not only experiences higher respiration rate but also higher ethylene production as compared to lower oxygen and higher dioxide conditions [45,46]. 

**Figure 4:**
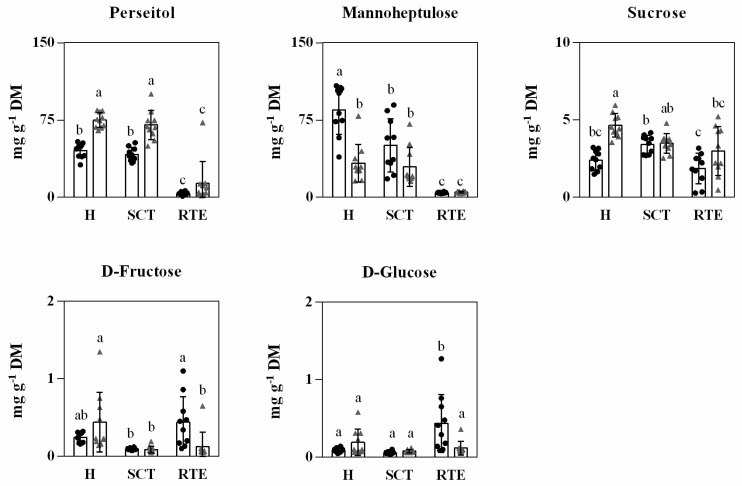
Abundance of main sugars in Hass avocado from two countries, Spain (black dots) and Chile (gray triangle) at harvest (H), after supply chain type (SCT) and at the ready-to-eat stage (RTE). Each column represents an average of 10 biological replicates and the standard deviation (SD) is represented by bars. Different letters indicate significant differences (*p* < 0.05) determined by ANOVA, followed by Tukey’s test.

Previous results of Hernández et al. [45] reported much lower metabolic activity evidenced as a complete firmness retention of avocado cv. Hass stored for 30 d in CA (similar conditions to the long-distance supply chain of this study) compared to regular air storage at 5 °C for 30 d, where enzyme action was on-going. Significant reductions of both sugars were observed at the ready-to-eat stage, reaching similar values in fruits from both origins and confirming the role of these two sugars as respiratory substrates in avocado (Figure 4). 

The C6 sugars instead only showed significant differences between origins at the ready-to-eat stage (RTE), the content of glucose and fructose being higher in Spanish avocados. Previous studies have reported differences in the content of C7 sugars among avocados from different origins, as the harvest season advances (early, middle, and late harvest fruit) and from harvest to edible ripeness [18,19,20]. Recently, Ramos-Aguilar et al. [47] reported values for the main sugars of 3.88, 4.98, 0.09, 0.34, 0.93, and 0.25 g kg^−1^ DW for fructose, glucose, sucrose, mannoheptulose, perseitol, and volemitol, respectively, at the mid of the harvesting season in Mexican Hass avocado fruits at edible ripeness. At the ready-to-eat stage, the short supply chain (Spanish avocados) presented values of 0.44, 0.44, 1.86, 4.4, and 3.5 g kg^−1^ DW of fructose, glucose, sucrose, mannoheptulose, and perseitol, respectively. In comparison, the long-distance supply chain (Chilean avocados) presented at the ready-to-eat stage values of 0.12, 0.12, 2.99, 5.01, and 13.69 g kg^−1^ DW of fructose, glucose, sucrose, mannoheptulose, and perseitol, respectively.

**Figure 5:**
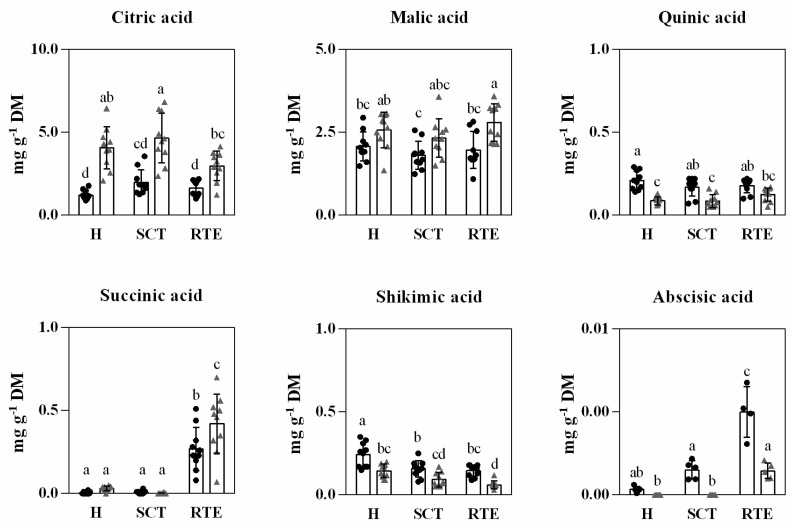
Abundance of main organic acids in Hass avocado from two countries, Spain (black dots) and Chile (gray triangle) at harvest (H), after supply chain type (SCT) and at the ready-to-eat stage (RTE). Each column represents an average of 10 biological replicates and the standard deviation (SD) is represented by bars. Different letters indicate significant differences (*p* < 0.05) determined by an ANOVA followed by Tukey test.

Organic acids present in avocado mesocarp have not received much attention in the past [4]. Polar metabolite analysis based on GC-MS mainly revealed citric, malic, quinic, succinic, and shikimic acids in Hass avocado mesocarp (Figure 5). Higher concentrations of citric acid at harvest were found in Chilean Hass avocados (4.1 g kg^−1^ DW) compared to Spanish avocados (1.2 g kg^−1^ DW) with significant variations from harvest to edible ripeness only observed in Chilean avocados (Figure 5). Defilippi et al. [48] found a similar behavior for citric acid from physiological maturity to edible ripeness of Hass avocado, but a significant decrease of malic acid as ripening proceeded. Instead, our results showed that malic and succinic acids were present in similar amounts in avocados of both origins at harvest and only succinic acid significantly increased at the ready-to-eat stage with higher values of 0.42 g kg^−1^ DW in Chilean Hass avocados compared to Spanish Hass avocados (0.27 g kg^−1^ DW) (Figure 5). 

A recent study of Campos et al. [19] reported the content and trend of malic, quinic, succinic, and citric acids in coastal Peruvian Hass avocado and including different harvest times (early, middle, and late) from harvest to edible ripeness. Early harvested fruits (~25.5% dry matter content), containing similar dry matter content to the range in our study, showed a decreasing concentration trend of malic and citric acids from harvest, after prolonged cold storage (37 d at 7 °C) and at edible ripeness. Similar to our study, no significant changes in quinic acid and higher amounts of succinic acid at the ready-to-eat stage (Figure 5) were reported by Campos et al. [19].

**Figure 6:**
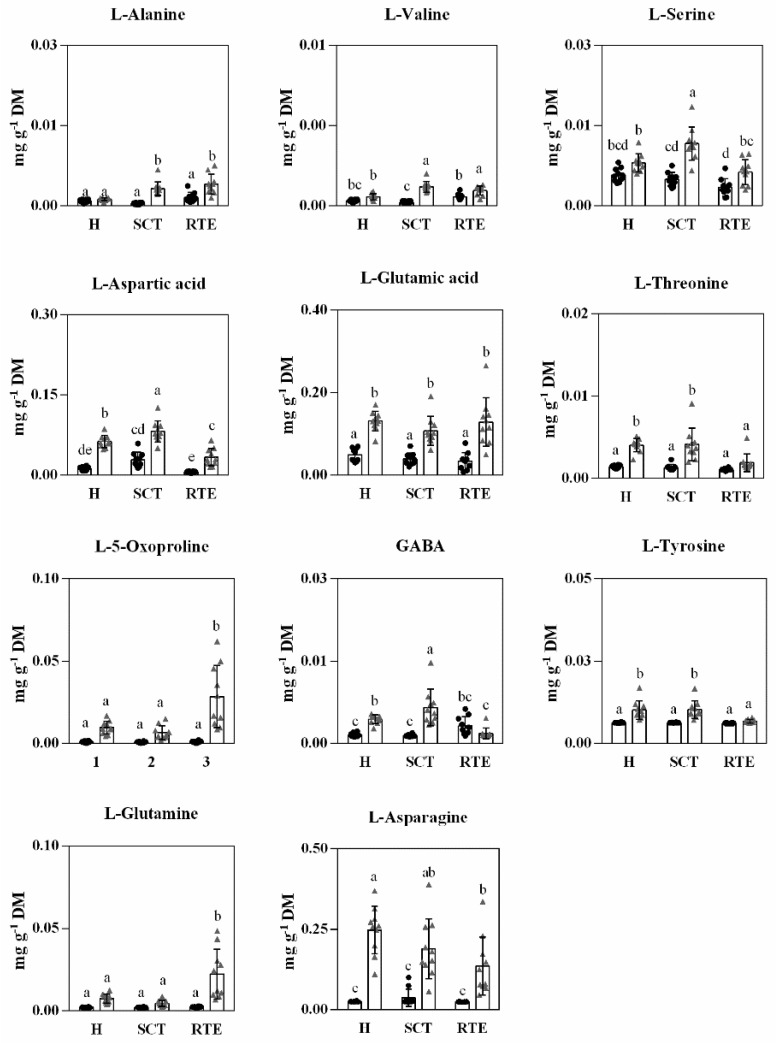
Abundance of amino acids in Hass avocado from two countries, Spain (black dots) and Chile (gray triangle) at harvest (H), after supply chain type (SCT) and at the ready-to-eat stage (RTE). Each column represents an average of 10 biological replicates and the standard deviation (SD) is represented by bars. Different letters indicate significant differences (*p* < 0.05) determined by an ANOVA followed by Tukey test.

Higher amounts of shikimic acid were reported at harvest in Spanish avocados (0.24 g kg^−1^ DW) compared to Chilean avocados (0.14 g kg^−1^ DW) and decreased from harvest to edible ripeness (Figure 5). Shikimic acid is a natural organic compound, and an important intermediate in the biosynthesis of lignin, aromatic amino acids (phenylalanine, tyrosine, and tryptophan), and most alkaloids of plants [49]. Its content and accumulation depend on different ongoing metabolic processes. The significant differences in shikimic acid concentrations between Spanish and Chilean Hass avocados from harvest onwards seem to be related to the differences in concentrations in aromatic amino acids (e.g., L-tyrosine) and total phenolics compounds in fruits of both origins, as shown below in Table 3 and Figure 6. At edible ripeness, the short distance supply chain avocados (Spanish) presented values of 0.27 g kg^−1^, 1.97, 0.15, 1.63, and 0.18 g kg^−1^ DW of succinic, malic, shikimic, citric, and quinic acids, respectively. For the long-distance supply chain, the values at edible ripeness were 0.42, 2.80, 0.06, 2.98, and 0.12 g kg^−1^ DW of succinic, malic, shikimic, citric, and quinic acids, respectively.

Ramos-Aguilar et al. [47] reported for avocados harvested at the mid of the harvesting season at edible ripeness, contents of 44.10, 1.16, 4.48, 10.14, and 0.33 g kg^−1^ DW of succinic, quinic, malic, citric, and ascorbic acids, respectively. Our lower values were obtained for succinic, quinic, malic, and citric acid, respectively (Figure 5), for early harvest avocado fruit.

Abscisic acid (ABA), a phytohormone, differed in content only after simulation of the respective different distance and conditions of the supply chain (short vs. long). For instance, the short distance supply chain (10 d at 6 °C) presented a higher content of ABA than the long-distance supply chain (30 d at 5 °C and 4 kPa O_2_ and 6 kPa CO_2_). At edible ripeness, the values increased for both supply chains with higher contents at the short distance supply chain (4 mg kg^−1^ DW). ABA has been suggested to play a role in the softening of avocado cv. Hass during ripening [50]. For avocado fruit mesocarp cv. Bacon, Vincent et al. [51] reported increases in ABA contents during ripening at 25 °C compared to fruits maintained at 4 °C but, compared to time zero, ABA also tended to increase during cold storage. In this study, the conditions of the long-distance supply chain that included controlled atmosphere in addition to cold storage resulted not only in lower concentrations of ABA after simulation of the supply chain but also at edible ripeness. 

Free amino acids in avocado mesocarp have also received little attention in the past [4] and no studies have evaluated their evolution from harvest to edible ripeness. Hass avocado mesocarp has been reported to contain 2% protein [6]. Free amino acids detected and quantified in this study corresponded to L-alanine, L-valine, serine, L-aspartic acid, L-glutamic acid, L-threonine, L-5-oxoproline, GABA, asparagine, L-tyrosine, and L-glutamine. Most of these amino acids were present in higher concentrations at harvest in Chilean Hass avocados (Figure 6). The metabolism of amino acids provides precursors for protein synthesis, for respiration processes and for a range of specialized metabolites including synthesis of secondary metabolites [52]. The long-distance supply chain (30 d in cold storage and CA) resulted in higher contents of L-alanine, L-valine, L-aspartic, GABA, and serine than the short distance supply chain (Spanish, 10 d in RA); several of these amino acids maintained higher concentrations even at the ready-to-eat stage (Figure 6). 

Previous studies in different fruit species have reported accumulation of amino acids during cold storage and their contents associated to conferring resistance to chilling injury [53]. However, under conditions of hypoxia such as the CA conditions of the long-distance supply chain, in addition, previous studies have reported that nitrogen metabolism is highly affected by oxygen deprivation with amino acids linked to the TCA cycle (main components of the respiration metabolism) changing their contents under oxygen stress compared to regular air conditions. For instance, the accumulation of GABA and alanine have been reported to be a common response of plants to hypoxia [54]. In addition, in apples stored in CA conditions, the contents of alanine, asparagine, GABA, proline, serine, and threonine were modulated by low oxygen stress [55]. Almost all these amino acids increased in concentration in the long-distance supply chain. Especially GABA and alanine increases are important adaptive processes, allowing for carbon and nitrogen storage, and, in addition, acting as osmo-protectants [53]. 

Amino acids L-5-oxoproline and L-glutamine significantly increased at the ready-to-eat stage only in Chilean Hass avocados, whereas GABA, L-aspartic, L-threonine, and L-tyrosine tended to decrease at the ready-to-eat stage mostly for Chilean Hass avocados (Figure 6). In addition, a significant reduction of L-tyrosine at edible ripeness was only observed in Chilean avocados, most likely because part of it is used for the synthesis of other intermediates used during respiration and derived to the synthesis of phenolic compounds [4]. In general, most of the amino acids detected and quantified in this study were present in higher concentrations at edible ripeness in the long-distance supply chain or Chilean avocados (Figure 6). 

Primary metabolites, especially sugars, organic acids, and amino acids act as respiratory substrates and are directly involved in the ripening of the fruit and postharvest performance and life. The results of this section revealed that the conditions of the chain (different for short and long-distance transport) evaluated the impact of the profiles of sugars, organic acids, or amino acids in addition to fruit origin, which is largely influenced by the growing conditions (Spanish vs. Chilean). The assumed belief that the longer the transport distance, the higher the negative impact on nutritional and functional compounds, can be controversial, since fruit origin as well as adequate transport conditions seems to be determinant of the fruit’s nutritional and functional components at the ready-to-eat stage. The conditions of the chain (regular air cold storage vs. cold storage plus controlled atmosphere conditions) play a critical role in maintaining and triggering the synthesis of several of the metabolites analyzed in this study.

### 3.4. Differences in Secondary Metabolites and In Vitro Antioxidant Capacity in Hass Avocado Fruit from Different Origins and Distance and Condition Supply Chains

Hass avocado has been reported as an important source of phenolic compounds not only in the peel but also in the mesocarp [19,21,47,52]. Differences in total phenolic content at harvest and after supply length simulation were observed between Spanish (1.59 and 1.42 g GAE kg^−1^ DW, respectively) and Chilean (1.42 and 0.94 g GAE kg^−1^ DW, respectively) Hass avocado mesocarp, but leveled in content at the ready-to-eat stage (0.90 and 0.94 g GAE kg^−1^ DW) (Table 3). Campos et al. [19] reported similar values of TPC content at harvest for Peruvian Hass avocados harvested at the mid of the harvesting season (1.58 g GAE kg^−1^ DW). This value was maintained after prolonged storage at 7 °C for 22 and 37 d and significantly increased at edible ripeness to 1.88 and 1.84 g GAE kg^−1^ DW, respectively. Lower values of TPC in Mexican Hass avocados harvested at the mid of the harvesting season (0.5 g GAE kg^−1^ DW) have been recently reported by Ramos-Aguilar et al. [47]. 

Similarly, Huamán-Alvino et al. [44] reported a content of 0.64 g GAE kg^−1^ DW for Peruvian Hass avocado harvested at the mid of the harvesting season grown in the Andean Region. Thus, the differences in the TPC content seem to be mainly related to the origin and, consequently, to the different growing conditions.

Regarding the comparison of phenolic profiles between Spanish and Chilean Hass avocados from harvest, after simulation of their respective supply chain and at edible ripeness, a total of 27 different phenolic compounds were detected and quantified including several hydroxybenzoic acid, epicatechin, syringic acid, p-coumaric, caffeic acid, and o-coumaric acid derivatives (Table 4). 

Some phenolics were only present at harvest in Hass avocados depending on the origin. At edible ripeness, phenolics that were only present in Spanish Hass avocados corresponded to hydroxybenzoic acid derivative 1 (HBA1), p-coumaric acid derivative 2 (PCAD2), caffeic acid derivative 2 (CAD2), o-coumaric acid derivative (OCAD), p-coumaric acid derivative 10 (PCAD10), unknown, p-coumaric acid derivative 11 (PCAD11), epicatechin derivative 4 (EPD4), epicatechin derivative 5 (EPD5), and epicatechin derivative 6 (EPD6). Similarly, phenolics only detected at edible ripeness in Chilean Hass avocado corresponded to p-coumaric acid derivative 1 (PCAD1), caffeic acid derivative 3 (CAD3), caffeic acid derivative 4 (CAD4), p-coumaric acid derivative 9 (PCAD9), and epicatechin derivative 3 (EPD3). Total phenolics by means of UPLC-DAD analysis resulted in a higher content at edible ripeness in Spanish avocados (17.10 ± 3.14 mg GAE 100 g^−1^ DW) compared to Chilean Hass avocados (10.65 ± 2.05 mg GAE 100 g^−1^ DW). Even though the quantitative data differed between quantification of total phenolic compounds by spectrophotometry and UPLC-DAD (Table 3 and 4, respectively), the same trend can be observed. The content decreased in 43–44% in the short distance supply chain (10 d at 6 °C in regular air) versus the long-distance supply chain (30 d at 5 °C and 4 kPa O_2_ and 6 kPa CO_2_) where a 24–30% increase in the total content was present at edible ripeness (Table 3 and Table 4). Campos et al. [19] reported for Peruvian avocados harvested at the mid of the harvesting season at harvest and after 22 cold storage at 7 °C, the presence of only four phenolic compounds: dihydrobenzoic acid glycoside, syringic acid glycoside, hydroxybenzoic acid glycoside, and sinapic acid glycoside. Not only the TPC content increased in that study at edible ripeness, but also the phenolic profile presented 19 different compounds that included the synthesis of significant amounts of phenolic acids, p-coumaric, caffeic, and their derivatives. Our study revealed the presence of eight and five different phenolic compounds at harvest in Spanish and Chilean Hass avocados, respectively (Table 4), belonging to hydroxybenzoic acid, epicatechin, o-coumaric acid, p-coumaric derivatives, and an unknown compound in Spanish avocados, while Chilean avocados only presented at harvest hydroxybenzoic acid and epicatechin derivatives. The supply chain (short vs. long-distance) only affected the content of some phenolics present at harvest but not the profile. For instance, after simulation of the short distance supply chain for Spanish avocados, the contents of syringic acid derivative (SAD) and hydroxybenzoic acid derivative 3 (HBA3) increased, but the contents of p-coumaric acid derivative 11 (PCAD11) and epicatechin derivatives 4 and 5 (EPD4 and EPD5) decreased. Simulation of the long-distance supply chain for Chilean Hass avocados resulted in decreases in the contents of epicatechin derivative 1 (EPD1), hydroxybenzoic acid derivative 2 and 3 (HBA2 and HBA3), and increased of epicatechin derivative 3 (EPD3) (Table 4). A recent study by Ramos-Aguilar et al. [47] reported the main phenolics synthesized at edible ripeness, in the mesocarp of Mexican Hass avocados harvested at the mid of the harvesting season: chlorogenic acid, sinapic acid, procyanidin B1, tyrosol, and homovanillic acid. No presence of epicatechin or derivatives was reported. Thus, the origin or the production conditions influence the content and profile of phenolic compounds at harvest and, together with the influence of the supply chain (conditions), will determine the final content and profile of phenolics at edible ripeness. 

The results of the H-AC and L-AC at the different evaluated stages for both supply chains are shown in Table 3. The H-AC trends for the short and long-distance and condition supply chains at different stages differed significantly. For instance, the short distance supply chain presented a decreasing trend of H-AC from harvest to edible ripeness (60% reduction), whereas that decrease was less pronounced in the long-distance supply chain (8%). At the ready-to-eat stage, average values of H-AC for the Spanish and Chilean avocados corresponded to 1.73 and 2.92 mmol TE kg^−1^ DW, respectively. No significant changes after both supply chain simulations were observed. However, at edible ripeness, contrary to previous works [19] that reported a significant increase in samples after 22 d cold storage but not after 37 d cold storage with values of 25.59 and 20.06 mmol TE kg^−1^ DW, in our study there was a significant decrease. Villa-Rodríguez et al. [21] reported a significant increase of H-AC at edible ripeness as well as an increase of TPC. In this study, the TPC content for Spanish avocados decreased at edible ripeness as well as H-AC, while for Chilean Hass avocados the content did not significantly change, and H-AC only slightly decreased at edible ripeness (Table 3). Ramos-Aguilar et al. [47] for Mexican Hass avocados harvested at the mid of the harvesting season reported a value of 1.12 mmol TE kg^−1^ DW, lower than both values of this study. The lipophilic antioxidant capacity (L-AC) differed between origins with higher values for Spanish avocados. However, from harvest to edible ripeness the values did not significantly differ in both origins. Campos et al. [19] reported for coastal Peruvian Hass avocados harvested at the mid of the harvesting season higher values of L-AC at edible ripeness (3.16 and 2.69 mmol TE kg^−1^ DW, 22 d and 37 d after cold stored, respectively). 

### 3.5. Integration of All Data by Multivariate Statistical Approaches

**Figure 7:**
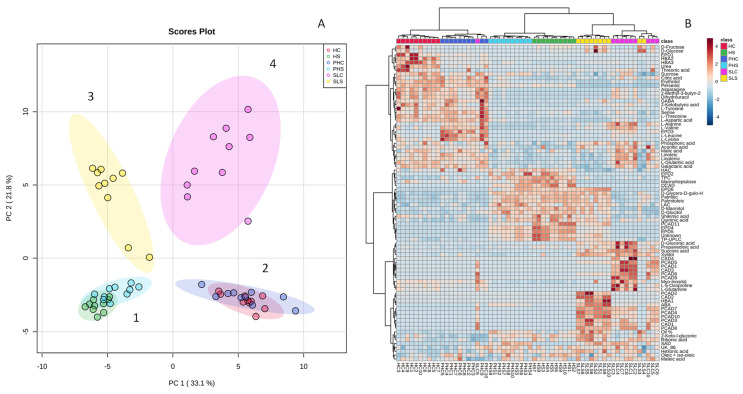
(**A**) Principal component analysis (PCA). (**A**) Score plot based on all primary and secondary metabolites measured of Hass avocado fruit representing a short (Spain) and long-distance supply chain (Chile): from harvest (HC and HS; for Chile and Spain), after the simulated transport chain (PHC and PHS; for Chile and Spain), and at the ready-to-eat stage (SLC and SLS for Chile and Spain). (**B**) Heat map based on cluster analysis using the Euclidean distance and Ward algorithm. Numbers 1-4 correspond to different clusters formed.

A principal component analysis (PCA) was performed considering all samples (at harvest, after supply chain simulation, and edible ripeness) for Spanish and Chilean Hass avocados harvested at the mid of the harvesting season and including all analyzed metabolites and parameters (Figure 7). The score plot (Figure 7A) was able to explain 54.9% of the total variance with the first two components. Four clear clusters can be observed. Cluster 1 gathered samples corresponding to harvest and after simulation of the short distance supply chain (6 °C for 10 d in regular air; Spanish avocados). Within this cluster, separation of samples corresponding to harvest and simulation of the supply chain can be observed, indicating that the conditions of the supply chain (10 d in RA at 6 °C) promoted slight changes in the pattern of certain metabolites. Cluster 2, in the opposite direction to cluster 1, gathered samples of Chile at harvest and after simulation of the long-distance supply chain (30 d at 5 °C and 4 kPa O_2_ and 6 kPa CO_2_); samples from both conditions overlapped, indicating that no major changes in metabolite composition take place during the long-distance supply chain conditions. Clusters 3 and 4 correspond to samples belonging to the short and long-distance supply chains at edible ripeness, respectively. These two clusters were clearly separated from each other, indicating that at edible ripeness, important changes in metabolite composition take place for both short and long-distance chains. Figure 7B corresponds to a cluster analysis based on Euclidean distance where samples are clearly discriminated, and the variables (primary, secondary, other polar metabolites, secondary metabolites, LAC, and HAC) are associated to each condition. Differences related to origin can be observed. Donetti and Terry [18] reported differences in fatty acid profiles as well as sugars among avocados from Spain, Chile, and Peru. Even within a country, due to differences in agroclimatic and growing conditions, differences in fatty acid profiles have been reported [19,43,44,56]. 

Increasing concerns related to transport distances and conditions of the supply chains and associated carbon emissions are emerging [8], but composition of the avocado in terms of primary and secondary metabolites at edible ripeness should also be considered to let consumers balance their decisions in terms of purchase intention. At edible ripeness, for instance, Spanish or short distance supply chain Hass avocados presented higher contents of glucose, fructose, LAC, oleic acid, shikimic, and quinic acids than Chilean Hass avocados. However, Chilean or long-distance supply chain avocados presented at edible ripeness higher contents of most of the amino acids detected, citric, malic and succinic acids, HAC, perseitol, myo-inositol, and xylitol (Figure 7B).

## 4. Conclusions

The LCA impact of Hass avocado, with a focus on the transport stage of the fresh supply chain from production centers in Chile (Quillota) and Spain (Malaga) and then distribution to several cities in Europe, suggested that road export from Spain to European capitals is the best option (0.14 to 0.22 kg CO_2_ eq/kg and 2.8 MJ to 4.3 MJ). When export from Chile is considered, the option of oceanic freight to European ports closer to final destinations are clearly better options (0.21 to 0.26 kg CO_2_ eq/kg and 3.8 MJ to 4.4 MJ) than exporting Chilean avocados via the Algeciras port, which increases the road transport to the final European capital destinations (0.34 to 0.43 kg CO_2_ eq/kg and 6.2 MJ to 7.8 MJ). However, it should be considered that the situation will be different when the avocados are transported from the destination ports in northern Europe to long-distance capitals in other European countries using road transport, which will increase CO_2_ emissions. 

Differences in primary and secondary metabolites and HAC and LAC were observed between Spanish and Chilean avocados related to fruit origin. Spanish avocados presented a higher content of oleic, palmitoleic, and palmitic acid than Chilean Hass avocados; instead, Chilean Hass avocados presented higher contents of linoleic and α-linolenic acids. The supply chain (distance and conditions) did not influence the contents and fatty acid profiles. Differences in the content of the two main C7 sugars present in avocado were detected at harvest between both origins; both decreased in different levels depending on the conditions of the supply chain (decreased more drastically in the short distance supply chain), but at edible ripeness, no significant differences were observed. Spanish avocados presented higher contents of quinic and shikimic acids at harvest. Both organic acids decreased in both chains, but values were higher at edible ripeness in the short distance supply chain. Chilean avocados presented higher contents of amino acids at harvest such as L-aspartic, L-glutamic, L-threonine, GABA, L-tyrosine, and L-asparagine.

The conditions of both supply chains (short vs. long-distance) influenced certain metabolites and HAC fate. A short distance supply chain (10 d at 6 °C) in regular air conditions resulted in a decrease of 60% of the HAC at edible ripeness while the conditions of the long-supply chain (30 d at 5 °C and 4 kPa O_2_ and 6 kPa CO_2_) only resulted in a 3% decrease of the HAC at edible ripeness. The long-distance supply chain promoted the accumulation of certain amino acids such as L-glutamine and L-5-oxyproline and accumulation of sugar alcohols (perseitol, myo-inositol, and xylitol). The short distance supply chain favored the biosynthesis of ABA. The profiles of phenolics at edible ripeness of both origins and supply chains were different mostly in terms of types of derivatives presents.

Our results suggest that besides the differences in the total carbon footprint of both transport supply chains (short and long-distance) and differences depending on the origin in terms of primary and secondary metabolites, the conditions of the supply chain itself (10 d in cold storage in regular conditions vs. 30 d cold storage + controlled atmosphere conditions) largely influence the fate of some metabolites that certainly will affect the pool of metabolites at edible ripeness. 

Further studies should combine LCA analysis from production to consumption with a complete metabolomics analysis at edible ripeness with different fruit origins to provide information on the environmental impacts on nutritional and functional properties of the fruits.

## Figures and Tables

**Table 1:** Distances used for the LCA calculation of the impacts of transportation of 1 kg of avocado cv. Hass fruit exported via different routes.

Country	Country of Origin to Port (by Road)	Distance to Export Country (Port to Port)	Main European Countries Importing Avocados	Distance by Road from Port to City
Chile	116 km (Quillota–San Antonio)	Rotterdam 18,020 km	The Netherlands/France	78 km to Amsterdam 456 km to Paris
		Marseille 17,011 km	France	776 km to Paris
		Portsmouth 17,309 km	United Kingdom	144 km to London
Chile	116 km (Quillota–San Antonio)	Algeciras 15,570 km	France	1938 km to Paris
			The Netherlands	2437 km to Amsterdam
			United Kingdom	2673 km to London
			Germany	2964 km to Berlin
			Spain	675 km to Madrid
Spain	--	0 km (Production in Malaga)	France	1802 km to Paris
			The Netherlands	2301 km to Amsterdam
			United Kingdom	2537 km to London
			Germany	2832 km to Berlin
			Spain	539 km to Madrid

All distances are converted to km and maritime routes are obtained from commercial information from http://www.ports.com (accessed on 15 January 2020). Distances covered by all terrestrial transports were determined using Google Maps (2021). Transport temperature corresponds to 5 °C.

**Table 2:** Oil and fatty acids content in Hass avocado from two countries (Spain and Chile) at harvest, after short and long-supply chains, and at the-ready-to-eat stage.

	Spain			Chile		
	Harvest	Short Supply Chain	Shelf Life—Ready-to-Eat	Harvest	Long Supply Chain	Shelf Life—Ready-to-Eat
**Oil (%)**	11.83 ± 2.00 ^a^	14.74 ± 1.82 ^b^	18.27 ± 2.91 ^c^	10.05 ± 0.57 ^a^	10.94 ± 0.65 ^a^	10.84 ± 1.13 ^a^
**Fatty acids**						
Palmitic	197.63 ± 17.46 ^a^	182.50 ± 23.10 ^a^	179.06 ± 23.88 ^a^	115.40 ± 10.69 ^b^	131.78 ± 13.64 ^b^	126.54 ± 23.57 ^b^
Palmitoleic	95.38 ± 8.79 ^a^	89.49 ± 16.11 ^a^	85.38 ± 13.46 ^a^	42.18 ± 3.75 ^b^	47.82 ± 6.10 ^b^	47.53 ± 7.86 ^b^
Oleic	511.20 ± 61.63 ^a^	485.32 ± 66.70 ^a,b^	470.12 ± 76.80 ^a,b^	435.17 ± 34.3 ^b^	505.08 ± 50.1 ^a,b^	487.84 ± 42.0 ^a,b^
Linoleic	134.96 ± 9.28 ^a^	131.84 ± 18.50 ^a^	120.95 ± 21.25 ^a^	169.44 ± 20.50 ^b^	189.65 ± 19.78 ^b^	194.93 ± 33.60 ^b^
α-linolenic	9.17 ± 0.93 ^a^	9.24 ± 1.76 ^a^	8.34 ± 1.70 ^a^	14.73 ± 2.15 ^b^	15.26 ± 1.80 ^b^	18.78 ± 3.56 ^c^

The results are expressed as the mean value of 10 avocados ± standard error (*n* = 10). Different letters in the same row indicate significant differences (*p* < 0.05) determined by an ANOVA followed by Tukey test. Fatty acids (g kg^−1^ oil).

**Table 3:** Total phenolic compounds, in vitro hydrophilic (HAC), and lipophilic antioxidant capacity (LAC) in Hass avocado from two countries (Spain and Chile) at harvest, after short and long-supply chain and at the ready-to-eat stage.

	Spain			Chile		
	Harvest	Short Supply Chain	Shelf Life—Ready-to-Eat	Harvest	Long Supply Chain	Shelf Life—Ready-to-Eat
TPC	1.59 ± 0.21 ^a^	1.42 ± 0.20 ^a^	0.90 ± 0.16 ^b^	0.72 ± 0.09 ^b^	0.94 ± 0.18 ^b^	0.94 ± 0.22 ^b^
HAC	4.31 ± 0.55 ^a^	4.02 ± 0.77 ^a,b^	1.73 ± 0.58 ^c^	3.16 ± 0.65 ^a,b^	4.22 ± 1.65 ^a^	2.92 ± 0.50 ^b^
LAC	1.46 ± 0.26 ^a^	1.72 ± 0.30 ^a^	1.48 ± 0.23 ^a^	0.94 ± 0.18 ^b^	1.04 ± 0.18 ^b^	1.10 ± 0.19 ^b^

The results are expressed as the mean value of 10 avocados ± standard error (*n* = 10). Different letters in the same row indicate significant differences (*p* < 0.05) determined by an ANOVA followed by Tukey test. Abbreviations: DW, dry weight; TPC, total phenolic content (g GAE kg^−1^ DW); HAC, hydrophilic antioxidant capacity (mmol TE kg^−1^ DW); LAC, lipophilic antioxidant capacity, mmol TE kg^−1^ DW; GAE, gallic acid equivalents; TE, trolox equivalents.

**Table 4:** Phenolic compound profile in Hass avocado from two countries (Spain and Chile) at harvest, after short and long-supply chain, and at the ready-to-eat stage. The results are expressed as the mean value of 10 avocados ± standard error (*n* = 10). (*) Quantified as p-Hydroxybenzoic acid. Different letters in the same row indicate significant differences (*p* < 0.05) determined by an ANOVA followed by Tukey test.

		Spain			Chile		
Peak Number	Phenolic Compound Assigned (mg GAE 100 g^−1^ DW)	Harvest	Short Supply Chain	Shelf Life—Ready to Eat	Harvest	Long Supply Chain	Shelf Life—Ready to Eat
1	Hydroxybenzoic acid derivative 1 (HBA1)	0.0 ^a^	0.0 ^a^	0.30 ± 0.16 ^b^	0.0 ^a^	0.0 ^a^	0.0 ^a^
2	Epicatechin derivative 1 (EPD 1)	0.0 ^a^	0.0 ^a^	0.0 ^a^	3.05 ± 2.42 ^b^	0.76 ± 0.30 ^a^	0.0 ^a^
3	Syringic acid derivative (SAD)	0.0 ^a^	0.11 ± 0.04 ^b^	0.11 ± 0.05 ^b^	0.0 ^a^	0.0 ^a^	0.11 ± 0.04 ^b^
4	Hydroxybenzoic acid derivative 2 (HBA2)	0.0 ^a^	0.0 ^a^	0.0 ^a^	1.03 ± 0.54 ^b^	0.15 ± 0.08 ^a^	0.0 ^a^
5	Hydroxybenzoic acid derivative 3 (HBA3)	0.13 ± 0.05 ^a^	0.80 ± 0.49 ^a^	0.87 ± 0.15 ^a^	4.21 ± 1.85 ^b^	1.05 ± 0.37 ^a^	0.90 ± 0.17 ^a^
6	*p*-Coumaric acid derivative 1 (PCAD1)	0.0 ^a^	0.0 ^a^	0.0 ^a^	0.0 ^a^	0.0 ^a^	1.20 ± 0.31 ^b^
7	*p*-Coumaric acid derivative 2 (PCAD2)	0.0 ^a^	0.0 ^a^	0.41 ± 0.24 ^b^	0.0 ^a^	0.0 ^a^	0.0 ^a^
8	*p*-Coumaric acid derivative 3 (PCAD3)	0.0 ^a^	0.0 ^a^	0.22 ± 0.10 ^b^	0.0 ^a^	0.0 ^a^	0.13 ± 0.07 ^b^
9	Caffeic acid derivative 1 (CAD1)	0.0 ^a^	0.0 ^a^	0.11 ± 0.05 ^b^	0.0 ^a^	0.0 ^a^	0.09 ± 0.03 ^b^
10	Epicatechin derivative 2 (EPD2)	0.36 ± 0.22 ^a^	0.42 ± 0.28 ^a^	0.0 ^b^	0.0 ^b^	0.0 ^b^	0.0 ^b^
11	*p*-Coumaric acid derivative 4 (PCAD4)	0.0 ^a^	0.0 ^a^	0.58 ± 0.09 ^b^	0.0 ^a^	0.0 ^a^	0.27 ± 0.08 ^c^
12	*p*-Coumaric acid derivative 5 (PCAD5)	0.0 ^a^	0.0 ^a^	0.04 ± 0.01 ^a^	0.0 ^a^	0.0 ^a^	0.12 ± 0.05 ^b^
13	*p*-Coumaric acid derivative 6 (PCAD6)	0.0 ^a^	0.0 ^a^	1.25 ± 0.21 ^b^	0.0 ^a^	0.0 ^a^	3.75 ± 1.16 ^c^
14	Caffeic acid derivative 2 (CAD2)	0.0 ^a^	0.0 ^a^	0.11 ± 0.03 ^b^	0.0 ^a^	0.0 ^a^	0.0 ^a^
15	*p*-Coumaric acid derivative 7 (PCAD7)	0.0 ^a^	0.0 ^a^	1.04 ± 0.13 ^b^	0.0 ^a^	0.0 ^a^	0.82 ± 0.11 ^c^
16	*p*-Coumaric acid derivative 8 (PCAD8)	0.0 ^a^	0.0 ^a^	0.14 ±0.03 ^b^	0.0 ^a^	0.0 ^a^	0.13 ± 0.07 ^b^
17	Caffeic acid derivative 3 (CAD3)	0.0 ^a^	0.0 ^a^	0.0 ^a^	0.0 ^a^	0.0 ^a^	0.03 ± 0.02 ^b^
18	Caffeic acid derivative 4 (CAD4)	0.0 ^a^	0.0 ^a^	0.0 ^a^	0.0 ^a^	0.0 ^a^	0.03 ± 0.00 ^b^
19	*p*-Coumaric acid derivative 9 (PCAD9)	0.0 ^a^	0.0 ^a^	0.0 ^a^	0.0 ^a^	0.0 ^a^	0.06 ± 0.03 ^b^
20	o-Coumaric acid derivative (OCAD)	0.12 ± 0.05 ^a^	0.08 ± 0.05 ^a^	0.0 ^b^	0.0 ^b^	0.0 ^b^	0.0 ^b^
21	Epicatechin derivative 3 (EPD3)	0.0 ^a^	0.0 ^a^	0.0 ^a^	0.22 ± 0.13 ^a^	7.96 ± 1.99 ^b^	2.87 ± 1.08 ^c^
22	*p*-Coumaric acid derivative 10 (PCAD10)	0.0 ^a^	0.0 ^a^	0.34 ± 0.10 ^b^	0.0 ^a^	0.0 ^a^	0.15 ± 0.04 ^c^
23	Unknown *	5.54 ± 2.46 ^a^	3.38 ± 1.32 ^a^	3.22 ± 1.33 ^a^	0.0 ^b^	0.0 ^b^	0.0 ^b^
24	*p*-Coumaric acid derivative 11 (PCAD11)	0.41 ± 0.15 ^a^	0.12 ± 0.05 ^bc^	0.24 ± 0.05 ^b^	0.0 ^c^	0.0 ^c^	0.0 ^c^
25	Epicatechin derivative 4 (EPD4)	7.29 ± 2.04 ^a^	4.51 ± 0.92 ^b^	2.21 ± 0.51 ^c^	0.0 ^d^	0.0 ^d^	0.0 ^d^
26	Epicatechin derivative 5 (EPD5)	15.72 ± 2.71 ^a^	8.23 ± 2.05 ^b^	5.05 ± 1.23 ^c^	0.04 ± 0.02 ^d^	0.0 ^d^	0.0 ^d^
27	Epicatechin derivative 6 (EPD6)	0.46 ± 0.07 ^ab^	0.43 ± 0.25 ^b^	0.69 ± 0.15 ^a^	0.0 ^c^	0.0 ^c^	0.0 ^c^
	**Total**	**30.03 ± 6.83 ^a^**	**18.09 ± 4.16 ^b^**	**17.10 ± 3.14 ^bc^**	**8.56 ± 3.73 ^d^**	**9.93 ± 2.08 ^cd^**	**10.65 ± 2.05 ^bcd^**

## Data Availability

The data presented in this study are available upon request from the corresponding author.
